# Artificial Intelligence Revolutionizing the Field of Medical Education

**DOI:** 10.7759/cureus.49604

**Published:** 2023-11-28

**Authors:** Suresh Narayanan, Rajprasath Ramakrishnan, Elantamilan Durairaj, Arghya Das

**Affiliations:** 1 Department of Anatomy, All India Institute of Medical Sciences, Madurai, Madurai, IND; 2 Department of Microbiology, All India Institute of Medical Sciences, Madurai, Madurai, IND

**Keywords:** machine learning, simulation, virtual learning, assessment, medical education

## Abstract

Medical education has ventured into a new arena of computer-assisted teaching powered by artificial intelligence (AI). In medical institutions, AI can serve as an intelligent tool facilitating the decision-making process effectively. AI can enhance teaching by assisting in developing new strategies for educators. Similarly, students also benefit from intelligent systems playing the role of competent teachers. Thus, AI-integrated medical education paves new opportunities for advanced teaching and learning experiences and improved outcomes. On the other hand, optical mark recognition and automated scoring are ways AI can also transform into a real-time assessor and evaluator in medical education. This review summarizes the AI tools and their application in medical teaching or learning, assessment, and administrative support. This article can aid medical institutes in planning and implementing AI according to the needs of the educators.

## Introduction and background

Artificial intelligence (AI) is a field of computer science that focuses on creating algorithms and software that imitate human thinking and decision-making [1]. The rapid emergence of AI in today’s world can be attributed to the advancement of sophisticated algorithms, affordable graphic processors, and extensive annotated databases [1]. In recent years, AI has emerged as an integral part of healthcare education and has been adopted by several medical institutions worldwide [2,3]. The use of AI in medical education is rampant in Western countries compared to developing countries. The difference could be lessened by more infrastructural support from medical institutions in the developing world. Creating awareness among medical educators and students about AI tools is essential to developing and implementing AI-based tools in medical education [4,5].

There are limited studies that summarize the available AI tools and how they can be effectively used in medical education [3]. Although the current studies focused on analyzing the trends of AI usage in medical education, very few studies have attempted to categorize the AI tools based on their uses for relevant stakeholders (e.g., students, faculty members, and administrators) [3,6].

## Review

AI in medical teaching and learning

AI can influence the student learning process in three ways: direct teaching (imparting knowledge to the student while playing the role of a teacher), support teaching (playing a supporting role and collaborating with students as they learn), and empowering the learner (multiple students can collaborate to solve a complex problem upon feedback provided by the teacher) [2,3,6]. Integrating AI tools in learning can enhance students' knowledge, skill development, and understanding of complex medical concepts. The subthemes of the use of AI include chatbots, intelligent tutoring systems (ITSs), virtual patients, gamification, and adaptive learning systems.

Chatbots

Chatbots are AI systems programmed to understand, process, and respond to human queries on specific input data by retrieving information from internet databases and generating human language using their advanced natural language processing (NLP) model [7]. Since most medical students own a smartphone or tablet, the chatbot systems can be easily deployed as a web application to aid learning. Chatbots have received considerable attention due to their ability to generate human-like text and engage users in an interactive conversation [8]. In the context of medical education, chatbots can efficiently automate time-intensive tasks, such as summarizing and evaluating research and medical literature [9]. Chatbots are primarily utilized as virtual teaching assistants to answer learners' queries and suggest resource materials. These can serve as preclinical interactive tutors and ward assistants in clinical settings to help students understand complex clinical scenarios and assist in developing decision-making skills [7]. The primary benefits of using chatbots include integrating content, quick access, allowing multiple users and immediate assistance [9,10]. Despite these, presently available chatbots can potentially create false references, a phenomenon described as “hallucination” [7]. Therefore, students should not rely on chatbots completely but use them as assistive tools only. Programming future chatbots specifically for medical education should be based on evidence-based resources that meet medical science standards and ethical values [7].

Intelligent Tutoring System

AI-based ITSs are adaptive instructional systems that try to emulate the benefits of one-on-one human tutoring [11]. These algorithms can analyze vast amounts of data regarding the student's past performance, preferences and learning style. Based on this data, the algorithms identify the learning gaps and create a personalized learning experience that can be adapted to the student's needs and progress. Essentially, ITS emulate a well-trained human tutor by interpreting the student's answers and appropriately responding to the learner in an adaptive learning pathway. The common themes of ITS use in medical education include teaching course content, diagnosing strengths or gaps in students' knowledge, providing automated feedback, curating learning materials based on student's needs, and facilitating collaboration between learners [12].

Virtual Patients

Virtual patients are interactive computer simulations of real-life clinical scenarios for health professions training and education. Virtual patients are programmed to exhibit realistic symptoms, respond to student’s interventions, and generate dynamic clinical experiences. The learner takes the role of a healthcare provider in acquiring information, suggesting differential diagnoses, medical management, and patient follow-up. These simulations can replicate various medical scenarios and present students with challenges they may face in real-life situations [13,14]. When interacting with virtual patients, medical students can practice their communication and clinical reasoning skills, creating an immersive and interactive virtual environment that mimics real-world scenarios [13,14].

Learning experiences with virtual patients can be made immersive using virtual reality (VR). VR is a software-based technology to create a three-dimensional simulated environment [15,16]. VR uses a head-mounted display or glasses to produce a computer-generated environment that feels realistic to the viewer. On the other hand, augmented reality (AR) improves a real-world environment by overlaying virtual components on a user's view of the real world via a smartphone or other device [17]. Incorporating these technologies enables learners to explore and engage with complex clinical scenarios in a way that makes the learning experience more enjoyable and effective.

Gamification

AI-driven games use data mining techniques to analyze the data collected while playing the game and improve the player's knowledge and skills [18]. They also offer a customizable and immersive experience that adjusts speed and difficulty based on the player's performance. Using game elements like points, badges, and leaderboards makes learning more enjoyable and engaging [18,19]. Gamification of the learning process increases student engagement, enhances collaborative efforts, and improves learning outcomes. They also provide opportunities for risk-free clinical decision-making and immediate feedback to the students, thereby becoming an integral part of undergraduate medical education [18,19].

Adaptive Learning System

When integrated with learning management systems (LMS), AI tools provide learners with the resources to attain mastery at their own pace [20]. These computer algorithms determine the learner's knowledge level and provide individualized instructional content to guide towards content mastery. The AI-based platforms drive the learners by appropriately spacing and sequencing the learning events, followed by specific remedial measures. These personalized and adaptive teaching methods improve learning efficacy and efficiency [20,21].

In addition to the above, AI-based tools may be beneficial for teaching in diagnostic disciplines like radiology, pathology, and microbiology. One such promising tool is content-based image retrieval (CBIR), used in teaching and research in radiology. CBIR helps by searching for images with contents similar to a reference image using information derived from the images [22]. Similarly, AI with machine learning is being used to diagnose microbial infections, which can be immense for training and educating technicians in microbiology [23]. On the other hand, the recent advancement in AI-based deep learning technologies focusing on cellular images can transform education in diagnostic pathology [24].

To summarize, AI can reduce the student burden in searching for reliable resources, provide a personalized adaptive learning experience with immediate feedback, and provide a simulation-based platform for a practical learning experience.

AI for assessment

AI can be utilized in various ways for assessment in medical education to avoid subjective bias. The scope of application includes evaluation of theoretical knowledge, assessment of diagnostic and procedural skills, and emergency response skills.

Optical Mark Recognition

Optical Mark Recognition (OMR) is the most common form, which has been practised for a long time for assessing objective questions like multiple choice questions and fill-in-the-blanks. This method helps evaluate a large number of answer sheets in a short time, and it also helps prepare a ranking system.

Automated Essay Scoring

Assessing written answer scripts is tedious as it is time-consuming and very subjective. After the development of AI's NLP, summative assessments can also be easily set using the automated essay scoring (AES) system. There are many studies conducted on the utilization of AES in various forms, and research is still going on [25]. AES scans the entire document and can understand the overall meaning and the concept even if written with grammatical errors [26]. This method, if incorporated into routine practice, can save time and human resources, and it can also keep track of the studying and knowledge pattern of the learner to give individualized feedback focussing on the areas of improvement.

Quizzing

As discussed earlier, gamification platforms can be used to create quizzes used for assessment. These quizzes can be funny and more engaging to the current students. The ability to track individual performance helps monitor the student's progress. These various gamification tools, like quizzes, puzzles, matching, drawing, etc., can be best utilized to promote self-assessment and in-class assessment practices.

Virtual Reality

Procedural skills can be assessed with VR, where a simulated patient will be presented in a virtual environment, and the learner is asked to do a procedure. Then, they will be assessed automatically by the steps they follow, and the system will give immediate feedback [16]. The establishment of VR labs is being started gradually among medical institutions. VR can be best employed in assessing the surgical skills of postgraduate students.

Simulation Assessment

On the other hand, without a virtual environment, the procedure can be done by an instructor in real life on a manikin, which is recorded and uploaded. AI can analyze and store the video in the form of consolidated data. If the same procedural video of the learner is uploaded, AI can compare the steps with preloaded video data and give comments and feedback about the procedure. This mode can be effectively utilized in situations of decreased student-teacher ratio.

Diagnostic Skill Assessment

As discussed earlier, AI teaches various clinical case scenarios as problem-based or case-based approaches. This teaching methodology can also be used as an assessment tool. If all the details of case data are already filled in, AI can formulate its own different sets of case scenarios to assess the learner's knowledge [27]. This will determine the higher order of diagnostic ability and critical thinking skills. AI can also create real-time scenarios based on the learner's knowledge level and adjust them dynamically so that the assessment will be individualized, and the lacunae and fields of improvement can be addressed individually.

Interpretation Skill Assessment

Machine learning is incorporated to interpret laboratory data accurately and detect abnormal values [28]. So, AI can be used to assess learner interpretation skills by developing specific AI algorithms with preloaded sets of extensive investigation data, including typical and atypical findings of various diseases. With the ability of AI to create its own sets of data for the same condition, individualized assessment can be done based on their cognitive levels.

Prescription Writing Skill Assessment

Medication errors are a significant preventable hazard in the health care system. Most of the errors happen in the transcription process, followed by prescription errors and then by errors in administration [29]. A medical professional must possess proper prescription writing skills. This can be evaluated by creating AI data sets and algorithms. Along with writing prescriptions, errors in prescriptions can also be assessed by developing incorrect prescriptions and asking the learner to evaluate the prescription and to comment on that with the help of preloaded comments using AI.

Emergency Response Skills Assessment

Response to an emergency is critical for a medical professional. Some studies show a lack of knowledge on managing anaphylaxis and safe blood transfusion skills [30,31]. This skill can be best acquired during an internship. So, assessing the knowledge of the steps to be done in emergency conditions, such as anaphylaxis management, precautions before and during blood transfusion, etc., is essential, and one has to be competent in all these skills. These emergency response skills can be assessed using AI with preloaded data on the acute management of these emergency conditions. Basic life support and advanced cardiac life support are the other skills best set using VR. Still, the steps alone can be evaluated using AI if the VR or the simulation facility is unavailable.

AI to aid academic medical researchers

AI tools can aid the researcher in collecting relevant research articles, identifying the knowledge gaps in the literature, formulating research questions, recommending appropriate statistical methods for the available data, creating a graphical representation of the data, and manuscript writing [32-35]. ResearchRabbit (Human Intelligence Technologies Incorporated, Seattle, WA, USA), an AI-based tool developed in 2021, enables researchers in medical institutes with novel ways to search for articles and writers, monitor research landscapes, and collaborate with researchers from other institutes. It strength of the extensive literature search lies in visualization maps based on network view (publications that are connected to one author) and timeline (publications plotted by the year) [32].

AI can also help researchers in analyzing vast amounts of data from patients’ treatment charts, laboratory reports, and textual feedback from students, and extract the relevant information needed for the research followed by sorting out the data in a structured manner for statistical analysis [36,37].

Artificial intelligence for administration in medical institutes

Administration in medical education involves the process of planning, organizing, directing, and controlling resources (human, financial, and physical) within an institution to achieve specific goals and objectives. It includes various activities such as curriculum development, faculty training, student assessment, and budgeting. Administrative tasks are also time and resource-consuming and sometimes involve the participation of academic staff along with administrative ones [38].

**Figure 1:**
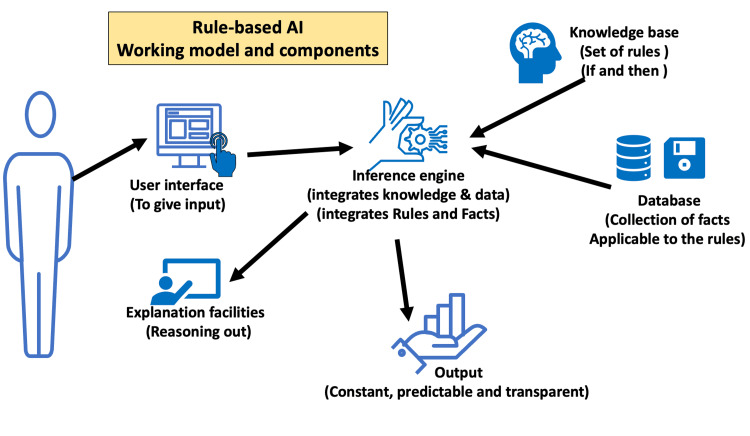
Basic working principle of rule-based artificial intelligence

**Figure 2:**
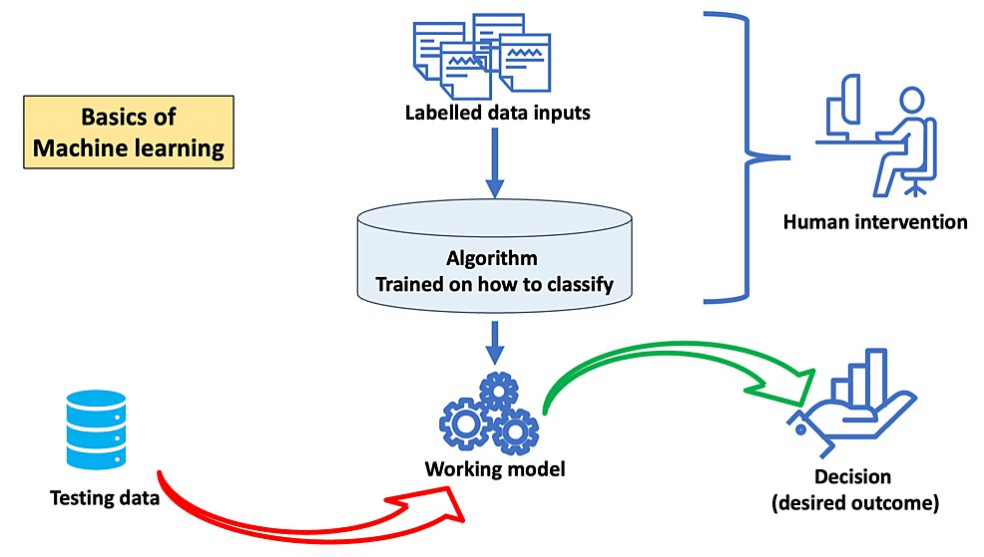
Basic working principle of machine learning

**Figure 3:**
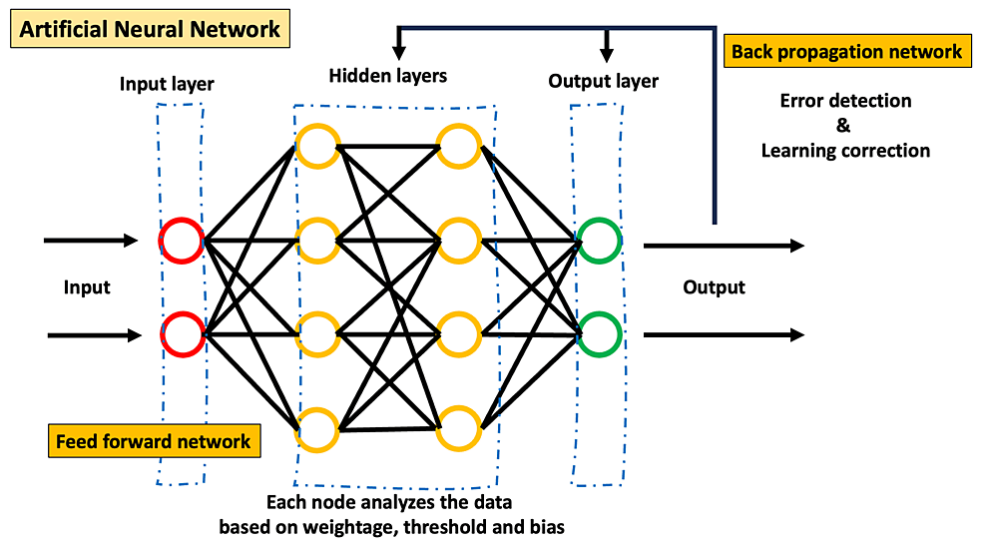
Basic working principle of artificial neural network

**Figure 4:**
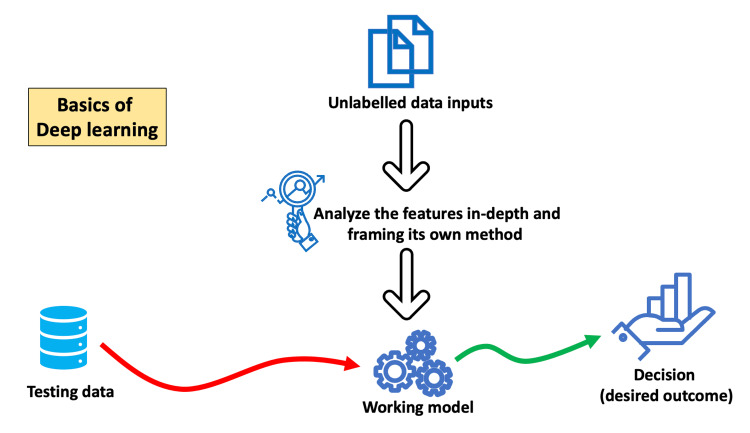
Basic working principle of deep learning

The application of AI in educational management and administration is still in its early stages but has already shown promising results. It can assist in automating plenty of administrative tasks and improve its overall efficiency [38]. AI primarily involves four technical approaches: rule-based, machine learning, neural networks, and deep learning. Figures 1-4 depict the basics of rule-based, machine learning, artificial neural networks, and deep learning concepts of AI.

Administrators of educational institutions are already using the former two approaches for decision-making in areas involving curriculum development, evaluation, and overall management. The latter two approaches are new to educational administrators. Deep learning allows multiple processing units and layers to process, learn, and represent data. Therefore, deep learning AI programs take enormous computing power and can outperform humans in identifying faces, recognizing speech, and many other tasks. Robotic process automation (RPA) software robots with machine-learning capabilities can automate admissions and billing functions. NLP, a branch of AI that helps computers understand and process human language, can automate workflows for administrative documentation, including the creation of transcripts and patient-case summaries [39]. The following are specific areas where AI is promising for medical administrators [2,40,41].

Recruitment and Admissions

AI can be used to analyze applicant data and identify candidates who are most likely to succeed in a given program. Scrutiny of many applications, segregating the applications, identifying fraudulent applications, and communicating with eligible candidates at multiple stages of admission/recruitment can be managed very efficiently by AI tools. By using machine learning algorithms to examine thousands of data points about each candidate, including their abilities, experience, education, and hobbies, recruiters can find the appropriate individuals more quickly and find those that best fit the institute's needs. The AI-powered large language model provides an efficient solution to evaluate candidates and make informed hiring decisions in a fraction of the time.

Curriculum Design and Review

Applying AI in trend analysis and identifying the information and abilities students will need in the future can help create new curricula and programmes. It reduces the time required to analyze different curricula, resolve multidimensional issues, enhance classification accuracy, and show a connection between the elements in curriculum assessment. The limited digitization of LMS for medical education, which is necessary for developing a curriculum map, is one potential explanation for the need for more use of AI in curriculum review. Designing a curriculum and identifying the program's desired outcomes are two ways to create one. The gap between the programme and the anticipated result may be determined using the expected result, and the curriculum can be developed to close that gap [42].

Management of Staff and Student Records

AI-powered systems can efficiently manage the records related to faculty and other staff and students. The payroll management, attendance and leave records management, scheduling exams, issuing admit cards, mark sheets, certificates, etc., can be seamlessly handled. AI can be valuable for additional activities like training and development, performance management, and workforce engagement. The increased automation in human resource management has brought machines and humans closer. AI and machine learning techniques have made studying and comprehending employee performance easier. Personalized learning and development programs would help employees improve their productivity by working on their weak areas [43].

Financial Aid and Student Services

AI can help institutions automate financial aid applications, identify students who may be eligible for scholarships or other aid forms, and provide personalized support to students.

Feedback Management

The feedback analytics can be significantly improved with the help of AI. It can guide in making intelligent decisions based on evidence.

Telemedicine

Telehealth uses information and communication technologies to transfer medical information to deliver clinical and educational services. Telemedicine for education and remote proctoring helps administrators educate colleagues remotely and collaborate with industry experts anytime, anywhere, without the risks and costs associated with travel. Care provision is becoming more complex due to increased innovation and discovery, going beyond the capabilities of any one healthcare provider. AI can support the development of knowledge of clinical processes. AI-enabled telehealth offers contributions in the form of quality improvement, enhancement of existing practice, and the instigation of new models of care and education [44].

Distance Education

Distance education is a type of teaching not limited by time or geography, allowing for real-time online and offline learning. Many countries have started implementing a 'dual approval system' for continuing medical education. This has enabled a multi-fold rise in certified professionals [45].

Virtual Inquiry System

These systems are developed for teaching hospitals and medical institutes. The approach is commonly utilized in preparing medical students and clinical thought evaluation. The software compiles hundreds of genuine patient data into individual cases, which are then studied with the help of AI. These data deal with a variety of clinical problems. Thus, medical students construct diagnosis and treatment plans for virtual patients using inquiry, simulated physical examinations, and supplemental examinations [45].

Budgeting and Resource Allocation

The analysis of current spending, their outcome, forecasting the expenses, budget proposals and allocation of resources can be very efficiently handled by AI systems. A study estimates AI in healthcare could save a net sum of 200 to 360 billion USD per year if tapped adequately in the United States alone [46]. Resources are always allocated by decision-making in budgeting procedures, and rational decision-making in resource allocation will likely increase investment returns. AI-based budgeting could present several options to enhance the allocation procedures. Identification of cause-and-effect links in value chains is a significant application of AI. By doing this, new insights can be identified to create optimized spending patterns [47].

Campus Safety

AI-powered surveillance systems can detect unusual behaviour and potential threats, alerting campus security personnel in real-time.

Miscellaneous

Also, the overall management involving maintenance of student-faculty-patient records systems, parent-teacher communication systems, information technology, LMS, cybersecurity, scheduling, budgeting, connected campuses, hospital information systems (HIS), hospital management and transportation can be geared up with the help of AI systems.

Challenges and prospects

Although AI is promising in different aspects of medical education, it has several drawbacks to universal adoption in medical educational institutes.

First, both machine learning and deep learning need enormous data sets to enable complex but accurate algorithms [48]. However, sharing data between medical institutes to generate large datasets is challenging due to organizational resistance, data privacy, and security concerns. A potential solution to this would be trust-building among institutions, accountability for data safety, and commitment towards the ethical use of AI.

Second, the next challenge is developing AI models to avoid biased outcomes following data collection. The use of an external dataset can validate results and reduce bias. Therefore, collaboration between institutions or even at the national level is crucial to allow for separate datasets for comparison [49].

Third, the apprehension regarding the replacement of staff by AI-based tools brings distrust and dislike to AI within an organization. However, this social fear of AI rendering teams and educators obsolete is a misunderstanding based on popular science fiction beliefs [49]. All cadres of staff need to be sensitized to the fact that AI would be practically impossible to successfully replace educators and administrators in an institute. Therefore, the application of AI should not be considered as a replacement for human resources but instead re-engineering jobs for efficiently delivering medical education services [50].

## Conclusions

Although AI has long been adopted in education, its application is still limited in medical institutions. As a futuristic approach to development, medical organizations should strategically orient educators and administrators about potential areas of AI in teaching, assessing, and management. As for any emerging technology, the success of AI in medical education will ultimately rely on organizational support and commitment by teachers and students to use the technology effectively within ethical boundaries.
